# Research on the relationship between key risk factors of university emergencies based on ISM-MICMAC

**DOI:** 10.1371/journal.pone.0317656

**Published:** 2025-02-03

**Authors:** Yufei Li, Xiaoyun Liu, Nanyan Hu, Xuexue Li

**Affiliations:** 1 School of Marxism, Wuhan University of Science and Technology, Wuhan Hubei, China; 2 School of Resource and Environmental Engineering, Wuhan University of Science and Technology, Wuhan Hubei, China; 3 Hubei Province Key Laboratory of Occupational Hazard Identification and Control, Wuhan University of Science and Technology, Wuhan Hubei, China; Hong Kong Baptist University, HONG KONG

## Abstract

In order to accurately analyze the interaction among the risk factors of university emergencies, firstly, based on the key performance indicator (KPI) concept, by initially extracting risk factors, identifying initial risk factors, and correcting initial risk factors, and the risk factors of emergencies in universities were divided into three categories: human factors, environmental factors, and management factors, and 18 key risk factors of emergencies in universities, including psychological problems, weak legal awareness and lack of safety awareness, were subdivided and determined. Secondly, the interpretive structure model (ISM) was used to construct a multi-layer hierarchical model of the relationship between university emergencies risk factors, clarified the hierarchical affiliation between the risk factors of university emergencies, divided the risk factors of university emergencies into surface direct factors, middle indirect factors and deep fundamental factors, and analyzed the coupling effect of various risk factors on university emergencies. Thirdly, combined with the matrix impacts cross-reference multiplication applied to a classification (MICMAC), the driving force and dependence force of each risk factor of university emergencies were obtained, and the risk factors of university emergencies were divided into spontaneous factors, independent factors, dependent factors, linkage factors, etc. 4 categories, and targeted governance and prevention measures were proposed for different types of risk factors. Finally, the research results will help universities to accurately identify, warn and manage the key risks of emergencies, and provide a reference for the management of emergencies in universities.

## 1 Introduction

In recent years, there has been an increasing number of emergencies in universities [1]. Emergencies in universities involve numerous stakeholders such as universities, teachers, and students, becoming a source of instability and risk for both the school and society. The risk factors of emergencies in universities include human factors, technology, environment, management, etc. These risk factors are intertwined and influence each other [2–4], making emergencies in universities uncertain. The formation mechanism is difficult to fully understand, and the evolution path is difficult to accurately predict. How to identify key risk factors of emergencies in universities, explore the interrelationships between risk factors of emergencies in universities, reveal the disaster mechanism of risk factors in emergencies in universities, analyze the evolution process of risk in emergencies in universities, formulate risk control measures for emergencies in universities, and thus prevent and reduce the risk of emergencies in universities has always been an important topic worthy of attention and research.

At present, experts and scholars are conducting research on risk identification, risk assessment, and risk control of emergencies in universities. In terms of risk identification for emergencies in universities, Chen et al. [5] took social security emergencies in ethnic universities as the research object, and used research and analysis to screen 22 types of scenario elements from three aspects: disaster causing bodies, disaster bearing bodies, and disaster resistant bodies that trigger emergencies. Based on the distribution of weight values, they identified and extracted key scenario elements for emergencies. Zhang et al. [6] used the analytic hierarchy process (AHP) to construct a food safety risk assessment index system for university canteens. They determined the weights of evaluation indicators at all levels, providing a basis for risk assessment and governance of university canteens.

In terms of risk assessment of emergencies in universities, Liu et al. [7] established an evaluation index system for emergency management capabilities of universities using the entire life cycle process, and evaluated the emergency management capabilities of universities by improving the matter element extension model. Song et al. [8] established a fire risk assessment index system for university dormitories from four aspects: people, objects, environment, and management. They combined the grey correlation method and D-S evidence theory to establish a fire risk assessment model for university dormitories.

In terms of risk warning for emergencies in universities, Lv et al. [9] divided the sources of emergency warning information in universities into internal and external warning information, established an analysis process for emergency warning information in universities, and preliminarily constructed a conceptual model for obtaining, preprocessing, storing, and analyzing emergency warning information in universities. Zhou et al. [10] used Delphi method to establish an indicator set for the early warning and evaluation of emergencies in universities, and integrated Delphi and AHP methods. In the early warning and evaluation stage, they used weighted set-valued statistics to propose a method for early warning and evaluation of emergencies in universities based on Delphi AHP and weighted set-valued statistics.

In terms of risk control for emergencies in universities, Wu [11] constructed a network public opinion monitoring index system for emergencies in universities based on questionnaire surveys and the Analytic Hierarchy Process, determined warning levels, and proposed network public opinion response strategies of “vertical blocking” and “horizontal cutting”. Deng et al. [12] analyzed the factors affecting the rescue capabilities of emergency rescue volunteers in universities, proposed a probability calculation model for the distributable range of emergency rescue volunteers and the rescue response process, and constructed an emergency rescue mode for university emergencies with students as the first responders.

In summary, experts and scholars have conducted research on emergencies in universities from different perspectives, providing useful references and guidance for risk management and governance of emergencies in universities. However, in the current research on the risk of emergencies in universities, the interrelationships between risk factors of emergencies in universities have not been considered, making it difficult to effectively measure the degree of influence between different risk factors, resulting in a lack of targeted and effective risk control measures taken. There are numerous risk factors for emergencies in universities, and each risk factor has a certain correlation with each other. Clarifying the relationship between risk factors is an important part of conducting risk assessment and decision-making for emergencies in universities.

Therefore, based on the ISM, a multi-level hierarchical model of the relationship between risk factors of emergencies in universities is constructed. Using MICMAC to analyze the degree of influence and interaction between risk factors of emergencies in universities, and propose corresponding control measures for different risk categories identified.

## 2 Determination of key risk factors for university emergencies

### 2.1 Concept of key risk factors

The KPI method was proposed by Italian economist Pareto, its core is to extract important and key indicators from numerous performance evaluation indicator systems, simplifying the evaluation of performance into the assessment of several key indicators [13]. KPI emphasizes the key indicators that have a critical impact on organizational performance, simplifying the numerous indicators used to measure work performance into several key indicators, which serve as the main body of organizational performance assessment and management. Key performance indicators are the hierarchical decomposition of organizational development goals. Through the integration of key performance indicators, individual performance behavior is aligned with organizational requirements to jointly achieve individual and organizational development goals, making key performance indicators the specific factors that truly drive the achievement of strategic goals. Due to the ability of KPI to capture key factors that have a decisive impact, its greatest advantage is that they can highlight the central work and focus on key tasks in the organizational performance evaluation process. For this reason, applying the KPI concept to the safety supervision of emergencies in universities, as long as key risk factors are identified as the focus of supervision, assessed and analyzed regularly or irregularly, and dynamic factor warnings are implemented, the key to risk prevention and control of emergencies in universities can be grasped [13].

**Fig 1:**
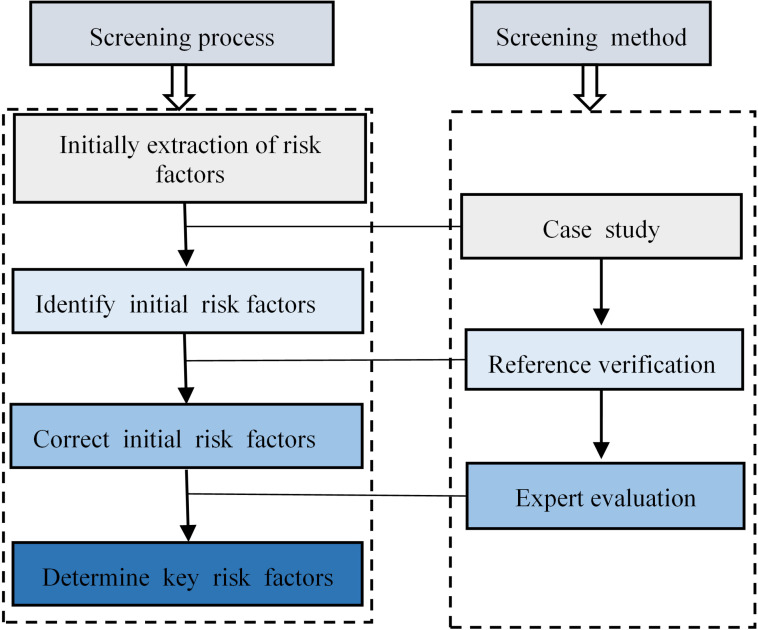
Chart for screening key risk factors in university emergencies.

The occurrence of emergencies in universities has the characteristics of insufficient precursors and strong destructiveness. Based on the KPI concept, key risk factors play a decisive role in the occurrence and development of emergencies in universities. The key to effective prevention and control of emergencies in universities is to screen out key factors. Timely decision-making and management based on the key factors obtained, changing the trajectory of the occurrence and development of emergencies in universities, curbing the spread of emergencies, and thereby reducing the harm or occurrence of emergencies in universities. Based on risk management theory, the key risk factors of emergencies in universities are defined as the factors that trigger the frequency of emergencies or have a significant impact on the severity of emergencies in universities. It is of great significance to study how to obtain the key risk factors of emergencies in universities for emergency management. The screening process for key risk factors in university emergencies is shown in Fig 1, which consists of four steps: initially extraction of risk factors, identification of initial risk factors, correction of initial risk factors, and determination of key risk factors.

The risk factors of emergencies in universities here specifically refer to the potential risk factors that cause emergencies in universities or the unstable factors that are prone to causing emergencies in universities. It focuses on the pre event risk management of emergencies in universities, and tends to enhance the prevention and early warning capabilities of emergencies in universities.

### 2.2 Initially extraction of risk factors

A comprehensive analysis of the risk factors of emergencies in universities is the prerequisite and foundation for determining the key factors of sudden incident risks in universities. According to the nature of the emergencies, emergencies in universities can be classified into suicide incidents, accidents, social security incidents, public health incidents, natural disasters, etc. 30 cases of emergencies that have occurred in universities in recent years were collected. The time, place, type, and consequence of the emergencies were described, and 25 risk factors were preliminarily extracted from real cases. The statistical information and risk factor extraction of university emergencies cases are shown in Table 1.

**Table 1:** Statistical information and risk factors extracted from 30 university emergencies cases in China.

No.	Time and place	Type	Consequence	Risk factor extraction
1	October 2005, Fujian	Natural disaster events	More than 80 students died.	uncontrollable natural disasters safety management system is not sound
2	July 2007, Hubei	Stampede accident	A girl was cut by fragments in the crowded area, and five or six people were slightly injured.	safety management system is not sound warning measures are not in place
3	November 2008, Shanghai	Fire accidents and incidents	Four girls jumped from the balcony of the 6th floor dormitory to escape and died on the spot.	insufficient promotion of safety knowledge lack of emergency training and drills incomplete investigation and management of hidden dangers weak safety awareness
4	January 2010, Guangdong	College student suicide incident	One student committed suicide.	emotional confusion unhealthy psychology insufficient support system
5	August 2010, Jiangsu	College student suicide incident	One student fell from a building and died.	excessive stress unhealthy mental state inadequate support system.
6	September 2010, Henan	Public health events	More than a hundred students were sent to the hospital for treatment, and 27 students with severe conditions experienced symptoms such as diarrhea, vomiting, and fever.	construction of safety equipment and facilities is weak investigation and treatment of hidden dangers are not thorough
7	April 2011, Hubei	College student suicide incident	One student committed suicide.	major family changes unhealthy mental state insufficient support system
8	June 2011, Shanxi	Social security incidents	One student died and three students were criminally detained.	lack of quality education insufficient promotion of safety knowledge weak legal awareness unhealthy psychological state
9	November 2011, Hubei	Social security incidents,	Dozens of students knelt down to petition the government.	investigation and governance of hidden dangers are not thorough surrounding environment of the campus is poor safety management system is not sound
10	Shanghai, April 2013	Social security incidents	One student died after being poisoned by someone else.	unhealthy psychological state poor interpersonal relationships lack of quality education inadequate safety management system
11	April 2013, Jiangsu	Abandoned laboratory explosion accident	The accident resulted in 1 death and 3 injuries.	safety management system is not sound investigation and governance of hidden dangers are not thorough warning measures are not in place safety awareness is not strong monitoring and warning system is not perfect
12	May 2013, Guangdong	Social security incidents	Hundreds of students from two schools are facing off.	lack of quality education safety awareness is not strong insufficient promotion of safety knowledge
13	June 2013, Jiangxi	College student suicide incident	One student committed suicide.	emotional confusion excessive stress unhealthy mental state inadequate support system
14	February 2014, Sichuan	Social security incidents	One student was sentenced to imprisonment for robbery and rape.	distorted outlook on life weak legal awareness lack of quality education
15	March 2014, Zhejiang	Public health events	Some students experience symptoms such as abdominal pain, diarrhea, and vomiting.	construction of safety equipment and facilities is weak investigation and treatment of hidden dangers are not thorough surrounding environment of the campus is poor promotion of safety knowledge is insufficient
16	December 2015, Beijing	Laboratory explosion accident	One postdoctoral fellow who was conducting an experiment died on the spot.	monitoring and early warning system is incomplete warning measures are not in place safety knowledge promotion is insufficient safety awareness is not strong
17	November 2015, Henan	Sudden death event	One student died suddenly during a physical examination on campus.	sports risks physical health problems inadequate safety management systems
18	June 2016, Shaanxi	Social security incidents	Causing the death of one student.	insufficient promotion of safety knowledge weak legal awareness unhealthy psychological state
19	August 2016, Jiangsu	Electric shock accidents	Two students died and one person was injured.	safety management system is not sound lack of emergency training and drills warning measures are not in place monitoring and warning systems are not perfect e.hidden danger investigation and governance are not thorough
20	April 2017, Fujian	College student suicide incident	One student committed suicide.	campus loan risks intentional destruction and external intrusion insufficient promotion of security knowledge inadequate support systems weak security awareness
21	August 2018, Heilongjiang	Drowning accident	One student died.	physical health problems insufficient promotion of safety knowledge incomplete investigation and management of hidden dangers weak safety awareness
22	December 2018, Beijing	Laboratory explosion accident	Three students who participated in the experiment died.	lack of strong safety awareness inadequate safety management system incomplete investigation and control of hidden dangers
23	July 2019, Hubei	Drowning accident	Two college students drowned and died.	insufficient promotion of safety knowledge.
24	October 2020, Jiangsu	College student suicide incident	One student fell from a building and died.	excessive stress unhealthy mental state poor interpersonal relationships
25	December 2020, Shandong	Social security incidents	One student was arrested for participating in theft.	insufficient promotion of safety knowledge lack of quality education.
26	March, 2021, Beijing	Laboratory explosion accident	Causing the death of one student.	insufficient promotion of safety knowledge weak safety awareness
27	March 2021, Henan	College student suicide incident	One self injured person died despite rescue efforts, resulting in two injuries.	unhealthy psychological state inadequate support system.
28	September 2021, Beijing	Traffic accident incidents	One student unfortunately passed away.	lack of safety awareness.
29	December 2021, Shaanxi	Public health events	Dozens of teachers and students were diagnosed with COVID-19.	spread of sudden public health emergencies insufficient promotion of safety knowledge inadequate safety management systems weak safety awareness
30	January 2022, Sichuan	Social security incidents	One student was raped.	insufficient promotion of safety knowledge weak safety awareness low level of quality

### 2.3 Identification of initial risk factors

Using reference research method, relevant literature on emergency events in universities was retrieved from databases such as China National Knowledge Infrastructure, Wanfang, and Web of Science. NoteExpress was used for reference retrieval, management, analysis, and discovery, and Excel was used to statistics and analyze the reference. After sorting and screening, 12 references with more citations and sufficient research were selected [5,14-24]. By using comparative analysis method, the risk factors of emergencies in universities identified in the reference were compared with the risk factors extracted from case studies. The comparison found that the risk factors obtained from case studies and reference research were basically consistent. Therefore, 25 risk factors for emergencies in universities can be considered as initial risk factors, as shown in Table 2.

**Table 2:** Initial risk factors for university emergencies.

No.	Initial risk factors
1	Safety management system is not sound
2	Inadequate warning measures
3	Insufficient promotion of safety knowledge
4	Lack of emergency training and drills
5	Inadequate investigation and management of hidden dangers
6	Lack of safety awareness
7	Emotional confusion
8	Unhealthy psychological state
9	Insufficient support system
10	Excessive stress
11	Weak construction of safety equipment and facilities
12	Major family changes
13	Lack of quality education
14	Weak legal awareness
15	Poor surrounding environment of campus
16	Poor interpersonal relationships
17	Monitoring and warning system is incomplete
18	Uncontrollable natural disasters
19	Distorted outlook on life
20	Sports risks
21	Physical health problems
22	Campus loan risk
23	Intentional destruction and external invasion
24	Spread of sudden public health emergencies
25	Low level of quality and competence

### 2.4 Correction of initial risk factors

Extracting key elements is essentially the process of hazard identification and risk analysis of emergencies. 10 experts in the field of safety were invited to revise and improve the initial risk factors, all of whom had been engaged in theoretical and practical research on safety management in universities, including 2 provincial-level safety production experts, 2 university professors, 2 personnel from education authorities, 2 personnel from university security departments, and 2 personnel from public security departments within the jurisdiction of universities. Firstly, based on expert interviews, opinions on improving the initial risk factors of emergencies in universities were obtained. Secondly, the AHP was used to determine the weights of initial risk factors, and the expert opinion clustering method was used to judge the degree of dispersion. For expert opinions with high dispersion, they could be considered as outliers and excluded. Thirdly, the initial risk factors were adjusted and suggestions were provided to experts for verification and confirmation. Fourthly, the risk factors for emergencies in universities that have been revised by experts were obtained. Finally, the risks of sports and campus loans were eliminated, and excessive stress, unhealthy psychology, poor interpersonal relationships, insufficient support systems, emotional confusion, distorted outlook on life, and other factors were integrated and classified as psychological problems.

### 2.5 Determine key risk factors

The key risk factors of emergencies in universities are the revised risk factors of emergencies in universities. Due to the large number of key risk factors in emergencies in universities, they lack systematicity. Therefore, the key risk factors of emergencies in universities were divided into human factors, environmental factors, and management factors, and ultimately identified as 18 key risk factors. The key risk factors of emergencies in universities are shown in Table 3.

**Table 3:** List of key risk factors for university emergencies.

Category	Risk factor	Number
Human factors (*A*)	Psychological problems	*S*_1_
	Weak legal awareness	*S*_2_
	Lack of safety awareness	*S*_3_
	Physical health problems	*S*_4_
	Low level of quality and competence	*S*_5_
Management factor (*B*)	Lack of quality education	*S*_6_
	Insufficient promotion of safety knowledge	*S*_7_
	Lack of emergency training and drills	*S*_8_
	The monitoring and warning system is incomplete	*S*_9_
	Inadequate warning measures	*S*_10_
	Inadequate investigation and management of hidden dangers	*S*_11_
	The safety management system is not sound	*S*_12_
	Weak construction of safety equipment and facilities	*S*_13_
Environmental factors (*C*)	Irresistible natural disasters	*S*_14_
	Spread of sudden public health emergencies	*S*_15_
	Intentional destruction and external invasion	*S*_16_
	Poor surrounding environment of campus	*S*_17_
	Major family changes	*S*_18_

## 3 Construction of ISM model for the relationship between key risk factors in university emergencies

The ISM qualitatively analyzes the hierarchical relationships between various risk factors of the research object from a system perspective, and is suitable for solving problems that contain numerous influencing factors and their interrelationships in the system. The formation of emergencies in universities is often the result of the coupling effect of multiple risk factors, and there is a certain correlation between these risk factors. The characteristics of emergencies in universities are consistent with the applicability of ISM. Therefore, it is considered to use ISM to explore the interrelationships and disaster mechanisms of risk factors for emergencies in universities.

### 3.1 Establishing adjacency matrix

The determination of the mutual influence relationship between various risk factors of emergencies in universities is the basis for conducting ISM modeling. There are often complex logical relationships between the risk factors of emergencies in universities. Based on the correlation of each factor, equation (1) is used to compare the risk factors of emergencies in universities in Table 3 pairwise, determine the binary relationship between the risk factors, and obtain the adjacency matrix ***A*** of the risk factors of emergencies in universities, as shown in Table 4.

**Table 4:** Aadjacency matrix of risk factors for university emergencies.

Factor	*S*_1_	*S*_2_	*S*_3_	*S*_4_	*S*_5_	*S*_6_	*S*_7_	*S*_8_	*S*_9_	*S*_10_	*S*_11_	*S*_12_	*S*_13_	*S*_14_	*S*_15_	*S*_16_	*S*_17_	*S*_18_
*S*_1_	0	1	1	1	0	0	0	0	0	0	0	0	0	1	1	1	0	0
*S*_2_	0	0	1	0	0	0	0	0	0	0	0	0	0	0	1	1	0	0
*S*_3_	0	0	0	0	0	0	0	0	1	1	1	1	1	1	1	1	0	0
*S*_4_	0	0	0	0	0	0	0	0	0	0	0	0	0	0	0	0	0	0
*S*_5_	0	1	1	0	0	0	0	0	1	1	1	1	1	1	1	1	0	0
*S*_6_	1	1	1	0	1	0	0	0	0	0	0	0	0	1	1	1	0	0
*S*_7_	0	1	1	0	0	0	0	0	0	0	0	0	0	1	1	1	0	0
*S*_8_	0	0	1	0	0	0	1	0	0	0	0	0	0	1	1	1	0	0
*S*_9_	0	0	0	0	0	0	0	0	0	1	1	0	0	1	1	1	0	0
*S*_10_	0	0	0	0	0	0	1	0	0	0	0	0	0	1	1	1	0	0
*S*_11_	0	0	0	0	0	0	0	0	0	1	0	0	0	1	1	1	1	0
*S*_12_	0	0	0	0	0	0	1	1	1	1	1	0	0	1	1	1	0	0
*S*_13_	0	0	0	0	0	0	0	0	1	1	0	0	0	1	1	1	0	0
*S*_14_	0	0	0	0	0	0	0	0	0	0	0	0	0	0	0	0	0	0
*S*_15_	0	0	0	0	0	0	0	0	0	0	0	0	0	0	0	0	0	0
*S*_16_	0	0	0	0	0	0	0	0	0	0	0	0	0	0	0	0	0	0
*S*_17_	0	0	0	0	0	0	0	0	0	0	0	0	0	0	0	0	0	0
*S*_18_	0	0	0	0	0	0	0	0	0	0	0	0	0	0	0	0	0	0

The adjacency matrix ***A*** of risk factors for emergencies in universities is:


$$A=\left[a_{\mathrm{ij}}\right]= \{\begin{array}{l} 1,\text{Impact}\\ 0,\text{No Impact} \end{array}$$ (1)

In the formula, *a*_*ij*_ represents the impact of risk factor *S*_*i*_ on factor *S*_*j*_ in a university emergency.

### 3.2 Generating reachable matrix

The adjacency matrix can only reflect the direct correlation between risk factors of emergencies in universities, and the occurrence of emergencies in universities often involves multiple risk factors that directly or indirectly affect each other. Therefore, in order to clarify the formation of emergencies caused by risk factors in universities, it is necessary to construct an reachable matrix that can express the degree of arrival of various risk factors in university emergencies based on the adjacency matrix, identify the indirect connections formed by the transmission of risk factors through complex paths, and comprehensively reflect the various relationships between risk factors in university emergencies. This can not only show the direct impact relationship between risk factors, but also reflect the indirect impact relationship between risk factors, which is conducive to revealing the disaster path of risk factors in university emergencies.

The expression for the reachable matrix ***Z*** of risk factors for emergencies in universities is:


$$Z\text{=}{\left(A\text{+}E\right)}^{k}={\left(A\text{+}E\right)}^{k-1}\neq {\left(A\text{+}E\right)}^{k-2}\neq \cdots \neq {\left(A\text{+}E\right)}^{2}\neq \left(A\text{+}E\right)$$ (2)

In the formula, ***E*** is the identity matrix. *k* is the number of operations.

Using MATLAB, the reachable matrix ***Z*** of risk factors for emergencies in universities was obtained from equation (2), as shown in Table 5. If the risk factor *z*_*ij*_ of the reachable matrix ***Z*** is 1, it represents that there is a reachable path between the risk factors *S*_*i*_ and *S*_*j*_. If the risk factor *z*_*ij*_ of the reachable matrix ***Z*** is 0, it means that there is no reachable path.

**Table 5:** Reachable matrix of risk factors for university emergencies.

Factor	*S*_1_	*S*_2_	*S*_3_	*S*_4_	*S*_5_	*S*_6_	*S*_7_	*S*_8_	*S*_9_	*S*_10_	*S*_11_	*S*_12_	*S*_13_	*S*_14_	*S*_15_	*S*_16_	*S*_17_	*S*_18_
*S*_1_	1	1	1	1	0	0	1	1	1	1	1	1	1	1	1	1	1	0
*S*_2_	0	1	1	0	0	0	1	1	1	1	1	1	1	1	1	1	1	0
*S*_3_	0	1	1	0	0	0	1	1	1	1	1	1	1	1	1	1	1	0
*S*_4_	0	0	0	1	0	0	0	0	0	0	0	0	0	0	0	0	0	0
*S*_5_	0	1	1	0	1	0	1	1	1	1	1	1	1	1	1	1	1	0
*S*_6_	1	1	1	1	1	1	1	1	1	1	1	1	1	1	1	1	1	0
*S*_7_	0	1	1	0	0	0	1	1	1	1	1	1	1	1	1	1	1	0
*S*_8_	0	1	1	0	0	0	1	1	1	1	1	1	1	1	1	1	1	0
*S*_9_	0	1	1	0	0	0	1	1	1	1	1	1	1	1	1	1	1	0
*S*_10_	0	1	1	0	0	0	1	1	1	1	1	1	1	1	1	1	1	0
*S*_11_	0	1	1	0	0	0	1	1	1	1	1	1	1	1	1	1	1	0
*S*_12_	0	1	1	0	0	0	1	1	1	1	1	1	1	1	1	1	1	0
*S*_13_	0	1	1	0	0	0	1	1	1	1	1	1	1	1	1	1	1	0
*S*_14_	0	0	0	0	0	0	0	0	0	0	0	0	0	1	0	0	0	0
*S*_15_	0	0	0	0	0	0	0	0	0	0	0	0	0	0	1	0	0	0
*S*_16_	0	0	0	0	0	0	0	0	0	0	0	0	0	0	0	1	0	0
*S*_17_	0	0	0	0	0	0	0	0	0	0	0	0	0	0	0	0	1	0
*S*_18_	0	0	0	0	0	0	0	0	0	0	0	0	0	0	0	0	0	1

### 3.3 Decomposing reachable matrix

From the reachability matrix, the reachable set *L*(*S*_*i*_) and antecedent set *D*(*S*_*i*_) corresponding to each risk factor can be obtained. The reachable set is the factor itself and the factors affected by it, representing the set of all risk factors reachable from factor *S*_*i*_. The antecedent set consists of the factors themselves and other influencing factors, representing the entire set of risk factors that can reach the *S*_*i*_ factor. The intersection of the reachable set and the antecedent set is the common set *C*(*S*_*i*_), denoted as:


$$C\left(S_{i}\right)=L\left(S_{i}\right)\cap D\left(S_{i}\right)$$ (3)

The reachable set, antecedent set, and common set of risk factors for emergencies in universities are shown in Table 6.

**Table 6:** The reachable set, antecedent set, and common set of risk factors for university emergencies.

Factor	Reachable set *L*(*S*_*i*_)	Antecedent set *D*(*S*_*i*_)	Common set *C*(*S*_*i*_)
*S*_1_	*S*_1_, *S*_2_, *S*_3_, *S*_4_, *S*_7_, *S*_8_, *S*_9_, *S*_10_, *S*_11_, *S*_12_, *S*_13_, *S*_14_, *S*_15_, *S*_16_, *S*_17_	*S*_1_, *S*_6_	*S*_1_
*S*_2_	*S*_2_, *S*_3_, *S*_7_, *S*_8_, *S*_9_, *S*_10_, *S*_11_, *S*_12_, *S*_13_, *S*_14_, *S*_15_, *S*_16_, *S*_17_	*S*_1_, *S*_2_, *S*_3_, *S*_5_, *S*_6_, *S*_7_, *S*_8_, *S*_9_, *S*_10_, *S*_11_, *S*_12_, *S*_13_	*S*_2_, *S*_3_, *S*_7_, *S*_8_, *S*_9_, *S*_10_, *S*_11_, *S*_12_, *S*_13_
*S*_3_	*S*_2_, *S*_3_, *S*_7_, *S*_8_, *S*_9_, *S*_10_, *S*_11_, *S*_12_, *S*_13_, *S*_14_, *S*_15_, *S*_16_, *S*_17_	*S*_1_, *S*_2_, *S*_3_, *S*_5_, *S*_6_, *S*_7_, *S*_8_, *S*_9_, *S*_10_, *S*_11_, *S*_12_, *S*_13_	*S*_2_, *S*_3_, *S*_7_, *S*_8_, *S*_9_, *S*_10_, *S*_11_, *S*_12_, *S*_13_
*S*_4_	*S*_4_	*S*_1_, *S*_4_, *S*_6_	*S*_4_
*S*_5_	*S*_2_, *S*_3_, *S*_5_, *S*_7_, *S*_8_, *S*_9_, *S*_10_, *S*_11_, *S*_12_, *S*_13_, *S*_14_, *S*_15_, *S*_16_, *S*_17_	*S*_5_, *S*_6_	*S*_5_
*S*_6_	*S*_1_, *S*_2_, *S*_3_, *S*_4_, *S*_5_, *S*_6_, *S*_7_, *S*_8_, *S*_9_, *S*_10_, *S*_11_, *S*_12_, *S*_13_, *S*_14_, *S*_15_, *S*_16_, *S*_17_	*S*_6_	*S*_6_
*S*_7_	*S*_2_, *S*_3_, *S*_7_, *S*_8_, *S*_9_, *S*_10_, *S*_11_, *S*_12_, *S*_13_, *S*_14_, *S*_15_, *S*_16_, *S*_17_	*S*_1_, *S*_2_, *S*_3_, *S*_5_, *S*_6_, *S*_7_, *S*_8_, *S*_9_, *S*_10_, *S*_11_, *S*_12_, *S*_13_	*S*_2_, *S*_3_, *S*_7_, *S*_8_, *S*_9_, *S*_10_, *S*_11_, *S*_12_, *S*_13_
*S*_8_	*S*_2_, *S*_3_, *S*_7_, *S*_8_, *S*_9_, *S*_10_, *S*_11_, *S*_12_, *S*_13_, *S*_14_, *S*_15_, *S*_16_, *S*_17_	*S*_1_, *S*_2_, *S*_3_, *S*_5_, *S*_6_, *S*_7_, *S*_8_, *S*_9_, *S*_10_, *S*_11_, *S*_12_, *S*_13_	*S*_2_, *S*_3_, *S*_7_, *S*_8_, *S*_9_, *S*_10_, *S*_11_, *S*_12_, *S*_13_
*S*_9_	*S*_2_, *S*_3_, *S*_7_, *S*_8_, *S*_9_, *S*_10_, *S*_11_, *S*_12_, *S*_13_, *S*_14_, *S*_15_, *S*_16_, *S*_17_	*S*_1_, *S*_2_, *S*_3_, *S*_5_, *S*_6_, *S*_7_, *S*_8_, *S*_9_, *S*_10_, *S*_11_, *S*_12_, *S*_13_	*S*_2_, *S*_3_, *S*_7_, *S*_8_, *S*_9_, *S*_10_, *S*_11_, *S*_12_, *S*_13_
*S*_10_	*S*_2_, *S*_3_, *S*_7_, *S*_8_, *S*_9_, *S*_10_, *S*_11_, *S*_12_, *S*_13_, *S*_14_, *S*_15_, *S*_16_, *S*_17_	*S*_1_, *S*_2_, *S*_3_, *S*_5_, *S*_6_, *S*_7_, *S*_8_, *S*_9_, *S*_10_, *S*_11_, *S*_12_, *S*_13_	*S*_2_, *S*_3_, *S*_7_, *S*_8_, *S*_9_, *S*_10_, *S*_11_, *S*_12_, *S*_13_
*S*_11_	*S*_2_, *S*_3_, *S*_7_, *S*_8_, *S*_9_, *S*_10_, *S*_11_, *S*_12_, *S*_13_, *S*_14_, *S*_15_, *S*_16_, *S*_17_	*S*_1_, *S*_2_, *S*_3_, *S*_5_, *S*_6_, *S*_7_, *S*_8_, *S*_9_, *S*_10_, *S*_11_, *S*_12_, *S*_13_	*S*_2_, *S*_3_, *S*_7_, *S*_8_, *S*_9_, *S*_10_, *S*_11_, *S*_12_, *S*_13_
*S*_12_	*S*_2_, *S*_3_, *S*_7_, *S*_8_, *S*_9_, *S*_10_, *S*_11_, *S*_12_, *S*_13_, *S*_14_, *S*_15_, *S*_16_, *S*_17_	*S*_1_, *S*_2_, *S*_3_, *S*_5_, *S*_6_, *S*_7_, *S*_8_, *S*_9_, *S*_10_, *S*_11_, *S*_12_, *S*_13_	*S*_2_, *S*_3_, *S*_7_, *S*_8_, *S*_9_, *S*_10_, *S*_11_, *S*_12_, *S*_13_
*S*_13_	*S*_2_, *S*_3_, *S*_7_, *S*_8_, *S*_9_, *S*_10_, *S*_11_, *S*_12_, *S*_13_, *S*_14_, *S*_15_, *S*_16_, *S*_17_	*S*_1_, *S*_2_, *S*_3_, *S*_5_, *S*_6_, *S*_7_, *S*_8_, *S*_9_, *S*_10_, *S*_11_, *S*_12_, *S*_13_	*S*_2_, *S*_3_, *S*_7_, *S*_8_, *S*_9_, *S*_10_, *S*_11_, *S*_12_, *S*_13_
*S*_14_	*S*_14_	*S*_1_, *S*_2_, *S*_3_, *S*_5_, *S*_6_, *S*_7_, *S*_8_, *S*_9_, *S*_10_, *S*_11_, *S*_12_, *S*_13_, *S*_14_	*S*_14_
*S*_15_	*S*_15_	*S*_1_, *S*_2_, *S*_3_, *S*_5_, *S*_6_, *S*_7_, *S*_8_, *S*_9_, *S*_10_, *S*_11_, *S*_12_, *S*_13_, *S*_15_	*S*_15_
*S*_16_	*S*_16_	*S*_1_, *S*_2_, *S*_3_, *S*_5_, *S*_6_, *S*_7_, *S*_8_, *S*_9_, *S*_10_, *S*_11_, *S*_12_, *S*_13_, *S*_16_	*S*_16_
*S*_17_	*S*_17_	*S*_1_, *S*_2_, *S*_3_, *S*_5_, *S*_6_, *S*_7_, *S*_8_, *S*_9_, *S*_10_, *S*_11_, *S*_12_, *S*_13_, *S*_17_	*S*_17_
*S*_18_	*S*_18_	*S*_18_	*S*_18_

According to the reachable matrix, the risk factors for emergencies in universities are classified into levels. The set of risk factors *N*_*i*_ for emergencies in level *i* universities is:


$$N_{i}=\{S_{j}|S_{j}\in P-N_{0}-N_{1}-\ldots -N_{i-1},C\left(S_{i}\right)=L\left(S_{i}\right),i=1,2,\ldots m\}$$ (4)

In the formula, *P* is the set of all risk factors, and the zero level *N*_0_ is set as an empty set.

According to equation (4), decomposing the reachable matrix ***Z*** yields the set of risk factors for emergency events in the first level universities as *N*_1_ = {*S*_14_, *S*_15_, *S*_16_, *S*_17_, *S*_18_}. By deleting the rows and columns corresponding to the first level risk factors, the same method can be used to obtain the set of emergency risk factors for the second level universities, which is *N*_2_ = {*S*_2_, *S*_3_, *S*_7_, *S*_8_, *S*_9_, *S*_10_, *S*_11_, *S*_12_, *S*_13_}. The set of risk factors for emergencies in the third level universities is *N*_3_ = {*S*_4_, *S*_5_}, the set of risk factors for emergencies in the fourth level universities is *N*_4_ = {*S*_1_}, and the set of risk factors for emergencies in the fifth level universities is *N*_5_ = {*S*_6_}.

### 3.4 Constructing ISM

**Fig 2:**
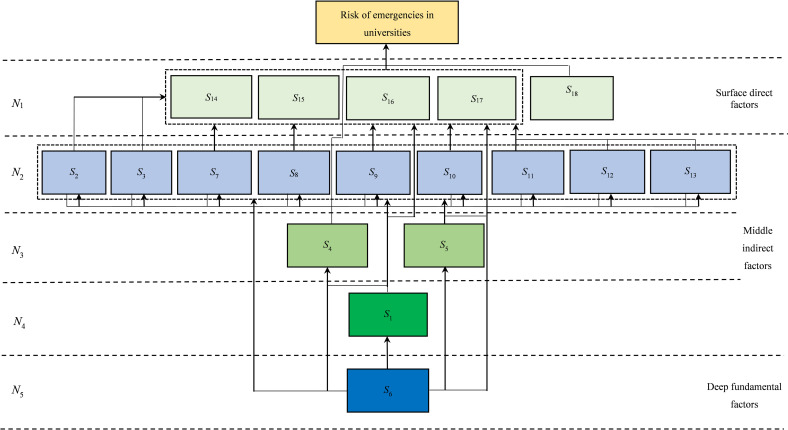
ISM of key risk factors for university emergencies.

In order to clarify the hierarchical relationship between the key risk factors of emergencies in universities and make their structural hierarchy clearer, based on the classification results of the five levels of emergencies risk factors in universities, the interrelationships between the risk elements of emergencies in universities are connected by arrows, and ISM of risk factors in university emergencies that reflects the interaction between risk factors is obtained. This is a multi-level hierarchical system consisting of five levels, where risk factors at different levels are closely related and form a logical chain of factors. The next level of factors directly or indirectly affects the previous level of factors. According to the position of risk factors in the explanatory structural model diagram, the 5 levels are divided into three regions: surface direct factors, middle indirect factors, and deep fundamental factors. Among them, the surface direct factors consist of the first layer, the middle indirect factors include the second, third, and fourth layers, and the deep fundamental factors are the fifth layer, as shown in Fig 2.

Surface direct factors directly affect the occurrence of emergencies in universities, which is the ultimate goal of managing and controlling emergencies in universities. The surface direct factors belong to environmental factors, including uncontrollable natural disasters *S*_14_, spread of sudden public health emergencies *S*_15_, intentional destruction and external invasion *S*_16_, poor campus environment *S*_17_, and major family changes *S*_18_. These five risk factors do not affect each other and are independent of each other, making them the most direct influencing factors for emergencies in universities.

Middle indirect factors have a driving effect on surface direct factors and are influenced by deep fundamental factors. It plays a transmission role in the explanatory structure model of key risk factors for emergencies in universities, transferring the driving influence from the lower level to the upper level factors, and cannot directly lead to the occurrence of emergencies in universities. Middle level indirect factors belong to human factors and management factors. Human factors include weak legal awareness *S*_2_, weak safety awareness *S*_3_, physical health problems *S*_4_, low level of quality *S*_5_, psychological problems *S*_1_. Management factors include insufficient promotion of safety knowledge *S*_7_, lack of emergency training and drills *S*_8_, imperfect monitoring and early warning systems *S*_9_, inadequate warning measures *S*_10_, incomplete hidden danger investigation and governance *S*_11_, inadequate safety management system *S*_12_, and weak construction of safety equipment and facilities *S*_13_. Human factors are distributed at three levels of middle indirect factors, while management factors are all located at the top level of middle indirect factors.

Deep fundamental factors do not directly affect emergencies in universities, but they have a fundamental impact on emergencies in universities and are decisive factors in such events. The underlying fundamental factor is the management factor, specifically the lack of quality education *S*_6_. Deep fundamental factors can directly or indirectly have a significant impact on upper level factors, and the key risk factors that should be emphasized in preventing emergencies in universities are the deep level impact.

### 3.5 Model analysis

In previous studies, the focus was often on analyzing a single factor that led to the occurrence of emergencies in universities. University emergencies have general characteristics such as multiple factors, multiple stages, dynamism, and uncertainty [25]. However, there is relatively little research on the coupling relationship of various risk factors in university emergencies from the perspective of risk coupling and explaining the mechanism of their occurrence. Based on risk coupling, the analysis of risk factors in emergencies in universities can identify the coupling relationships between risk factors, grasp the transmission of risk factors along different paths within or outside the university, form different risk evolution trends of emergencies in universities, and further clarify the complex occurrence process of emergencies in universities, providing a more comprehensive understanding of emergencies.

By analyzing the human factors, management factors, and environmental factors in the explanatory structure model of key risk factors for emergencies in universities, it is found that there are two types of coupling of risk factors for emergencies in universities: two factor coupling and three factor coupling. The coupling of two factors includes management factors environmental factors, management factors human factors, and human factors environmental factors. The coupling of management factors environmental factors is shown in *S*_6_ → *S*_17_, the coupling of management factors human factors is shown in *S*_6_ → *S*_4_, and the coupling of human factors environmental factors is shown in *S*_2_ → *S*_15_. The coupling of three factors includes management factors, human factors, and environmental factors, such as *S*_6_ → *S*_5_ → *S*_2_ → *S*_15_. Different coupling factors constitute different paths of emergencies in universities, all of which lead to the occurrence of emergencies in universities.

Mastering the coupling relationship between various risks of emergencies in universities is conducive to understanding the inherent connections between risk factors and the importance of risk factors, clarifying the disaster mechanism of risk factors, and then adopting effective risk management strategies and methods. On the one hand, by cutting off or reducing the coupling degree between strong coupling risk factors of emergencies in universities, the trajectory of emergencies can be changed to avoid or reduce the risk of emergencies in universities. On the other hand, in situations where unexpected events cannot be avoided in universities, reducing the probability of risk factors with high coupling to university emergencies, controlling the deepening and spread of the situation, and minimizing the harm caused by emergencies.

## 4 MICMAC analysis of key risk factors for university emergencies

### 4.1 MICMAC classification

MICMAC is a technique for analyzing the interaction relationships between various factors in a system. This method calculates the driving forces and dependencies of each factor through the relationship matrix between factors, classifies system elements, and achieves classification management and governance. Therefore, MICMAC is adopted to identify the driving and dependent factors in the risk factors of emergencies in universities, clarify the roles and positions of each risk factor in emergencies in universities, quantify the degree of influence between risk factors in emergencies in universities and the degree of influence of risk factors, determine the focus of risk management and intervention, and propose targeted prevention and control measures for different types of risk factors.

The driving force *D*_*i*_ is the degree to which a certain risk factor in a university emergency affects other risk factors in the system. It is the sum of the rows in the reachable matrix ***Z***, and its calculation formula is:


$$D_{i}=\sum_{j=1}^{n}a_{ij}(i=1,2,3\cdots ,n)$$ (5)

The degree of dependence of a certain risk factor in a university emergency on other risk factors in the system is called dependency *R*_*j*_, which is the sum of the columns of the reachable matrix ***Z***. Its calculation formula is:


$$R_{j}=\sum_{i=1}^{n}a_{ij}(j=1,2,3\cdots ,n)$$ (6)

In the formula, *a*_*ij*_ is a factor in the reachable matrix ***Z***.

According to equations (5) and (6), calculate the driving forces *D*_*i*_ and dependencies *R*_*j*_ of various risk factors for emergencies in universities, as shown in Table 7.

**Table 7:** Driving forces and dependence of risk factors for university emergencies.

Risk factor	*D*	*R*	Risk factor	*D*	*R*
*S*_1_	15	2	*S*_10_	13	12
*S*_2_	13	12	*S*_11_	13	12
*S*_3_	13	12	*S*_12_	13	12
*S*_4_	1	3	*S*_13_	13	12
*S*_5_	14	2	*S*_14_	1	13
*S*_6_	17	1	*S*_15_	1	13
*S*_7_	13	12	*S*_16_	1	13
*S*_8_	13	12	*S*_17_	1	13
*S*_9_	13	12	*S*_18_	1	1

**Fig 3:**
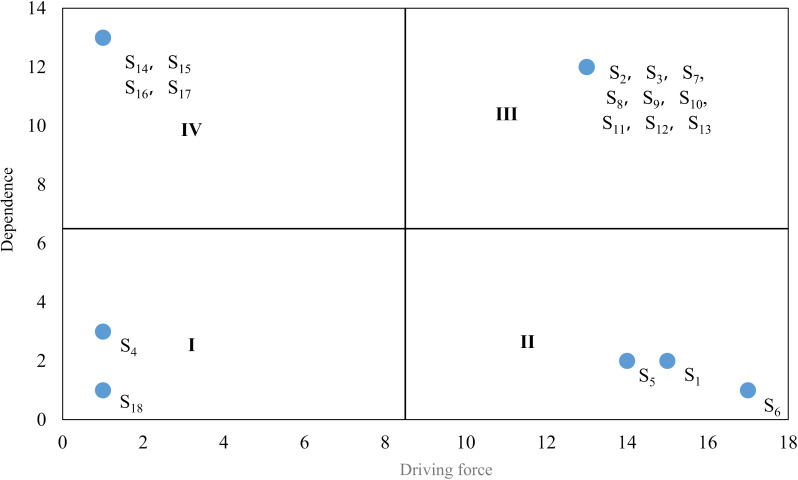
Quadrant diagram of driving force dependence of risk factors for emergencies in universities.

According to Table 7, with driving force as the horizontal axis and dependence as the vertical axis, draw a quadrant diagram of driving force dependence of risk factors for emergencies in universities, as shown in Fig 3. According to the driving force and dependence of each risk factor, the risk factors of emergencies in universities are divided into four categories: spontaneous factors in quadrant I, independent factors in quadrant II, linkage factors in quadrant III, and dependent factors in quadrant IV. The driving force of spontaneous factors is weak, and the dependence is weak. The strong driving force of independent factors and weak dependence. The driving force of dependent factors is weak, while the dependence force is strong. The driving force of linkage factors is strong, and the dependence is strong.

### 4.2 Result analysis

According to Fig 3, a detailed analysis of spontaneous factors, independent factors, linkage factors, and dependent factors is conducted to provide theoretical support for targeted management strategies.

The first type of factors with low driving force and dependence are called spontaneous factors, which are relatively independent and less susceptible to the influence of other factors. They have a small correlation with the system, but may have a strong impact on the system. When considering their constraining factors, they need to be considered separately.

The second type of factors with high driving force but low dependence are called independent factors. These factors are generally at the bottom of the ISM model because their driving force is high. Effective control of these factors can effectively alleviate the direct negative impact of other factors.

The third type of factors with high driving force and dependence are called linkage factors, which are generally located in the middle layer of the ISM model and play a transmitting role.

The fourth type of factors with low driving force but high dependence, which are usually at the top of the ISM model and can be controlled by controlling other factors [26].

(1) Spontaneous factors

Risk factors such as physical health problems *S*_4_ and major family changes *S*_18_ are spontaneous factors with weak driving force and dependence. These risk factors are relatively independent and not easily influenced by other risk factors, and have a relatively small impact on other risk factors. The correlation with the system is relatively small, but they have a direct impact on emergencies in universities and are important risk factors that cannot be ignored.

(2) Independent factors

Risk factors such as psychological problems *S*_1_, low level of quality *S*_5_, and lack of quality education *S*_6_ are independent factors with strong driving force and weak dependence, generally located at the bottom of the ISM. These factors are less affected by other factors, but have a greater impact on them, with strong conductivity, and are the core risk factors that trigger emergencies in universities. Due to the strong driving force and low dependence of the lack of quality education *S*_6_, it indicates that it is influenced by a few risk factors, but has a greater impact on the upper level risk factors of the ISM. Therefore, the focus should be on the lack of quality education *S*_6_.

(3) Linkage factors

Weak legal awareness of risk factors *S*_2_, weak safety awareness *S*_3_, insufficient promotion of safety knowledge *S*_7_, lack of emergency training and drills *S*_8_, incomplete monitoring and early warning system *S*_9_, inadequate warning measures *S*_10_, incomplete hidden danger investigation and governance *S*_11_, incomplete safety management system *S*_12_, weak construction of safety equipment and facilities *S*_13_ belong to linkage factors, with strong driving force and dependence, generally located in the middle layer of the ISM, easily influenced by other factors, and can be transmitted to other factors, playing a transmitting role.

(4) Dependency factors

The uncontrollable natural disasters *S*_14_, spread of sudden public health emergencies *S*_15_, intentional destruction and external invasion *S*_16_, and poor campus environment *S*_17_ are all dependent factors, with weak driving force and strong dependence. These types of risk factors are usually at the top level of the ISM, close to the final risk of university emergencies, formed by the accumulation of other risk factors, with a relatively small impact on other risks, and are the main manifestation of university emergency risk.

### 4.3 Management strategies

The risk management of emergencies in conventional universities mainly involves phased governance before, during, and after the event. Risk management should focus on prevention, and this risk control strategy involves a wide range of areas and is generally macro level. Especially before preventing emergencies in universities, multiple factors such as personnel, facilities, equipment, and environment need to be comprehensively considered, and the mutual influence between risk factors is not taken into account. The actual operation is difficult, and the pertinence and effectiveness are not strong. Using MICMAC analysis can identify priority governance factors, key governance factors, etc., and explore how to prevent and manage risk factors of emergencies in universities from the perspective of risk correlation

(1) Prioritize the governance of spontaneous factors

Spontaneous factors have strong independence, do not affect other risk factors of emergencies in universities, do not lead to the formation of risk chain effects, and are less affected by other risks. Compared to other risk factors, they are easier to control and have simpler governance. Therefore, spontaneous factors should be the first to be controlled in the management of risk factors for emergencies in universities, in order to reduce the risk of emergencies in universities.

(2) Key governance independent factors

Independent factors, due to their high driving force, may not directly trigger emergencies in universities, but they have a significant impact on the risk of emergencies in universities. Controlling independent factors can effectively alleviate the direct adverse effects on other risk factors, avoid a series of “butterfly effects”, and have a significant effect on the prevention and control of emergencies in universities. Therefore, it is necessary to focus on addressing the independent factors of emergency risks in universities.

(3) Block and weaken the linkage factors

The linkage factors will not directly lead to the risk of emergencies in universities, but are influenced by other risk factors of emergencies in universities. To block and weaken the transmission of linkage factors, on the one hand, the connection between linkage factors and upper level risk factors in the ISM can be controlled to avoid the occurrence of emergencies in universities. On the other hand, it is possible to control the underlying risk factors of linkage factors and prevent the risk of unexpected events in universities.

(4) Regulating and controlling dependent factors

The emergence of dependency factors is mostly the result of the interweaving and joint effects of other risk factors in university emergencies. The driving factors of dependency factors should be identified, and the risk of university emergencies should be reduced by strengthening personnel training and emergency preparedness. In addition, based on dependency factors, strengthen the research on the main types of emergencies in universities, grasp the characteristics and laws of different types of emergencies in universities, and adhere to classified management.

This study helps university managers identify key risk factors and the hierarchical relationships between risk factors in university emergencies, and gain a deeper understanding of the mechanisms by which risk factors influence each other and lead to the occurrence of university emergencies. Predicting and warning of emergencies in universities before they occur can, on one hand, prevent the occurrence of university emergencies through warning mechanisms. On the other hand, warning mechanisms can be used to identify potential risk factors and obtain efficient and accurate risk management plans and countermeasures.

## 5 Conclusions

(1) Based on the KPI concept, after preliminary extraction of risk factors, identification of initial risk factors, and correction of initial risk factors, the key risk factors of emergencies in universities are divided into human factors, environmental factors, and management factors. Finally, 18 key risk factors in three categories, including psychological problems, weak legal awareness, and weak safety awareness, are determined to comprehensively reflect the risk attributes of emergencies in universities. The combination of ISM-MICMAC method is adopted to explore the interrelationships between key risk factors of emergencies in universities, providing new ideas and scientific research methods for emergency management of emergencies in universities.
(2) The ISM of key risk factor relationships in universities emergencies was constructed, which is a multilevel recursive system containing five levels, reflecting the hierarchical relationships of risk factors, identifying the surface direct factors, middle indirect factors and deep fundamental factors, and analyzing the formation mechanism of analyzing college emergencies under the conditions of risk factor interactions with the existence of two-factor coupling and three-factor coupling paths of causation. The surface direct factors directly affect the occurrence of emergencies in universities. Middle indirect factors have a driving effect on surface direct factors and are influenced by deep fundamental factors. Deep fundamental factors do not directly affect emergencies in universities, but they have a fundamental impact on emergencies in universities and are decisive factors in emergencies.
(3) MICMAC is used to analyze the relationship between key risk factors in university emergencies, identify factors with dynamic and dependent characteristics in the system, determine the spontaneous factors, dependent factors, linkage factors, and independent factors of university emergency risk, and propose risk management strategies for university emergencies that prioritize the governance of spontaneous factors, focus on the governance of independent factors, regulate and control dependent factors, and block and weaken linkage factors. By improving risk response measures in advance, the likelihood of emergencies occurring in universities can be reduced, the impact of such university emergencies on teachers and students can be minimized, and the level of safety management in universities can be improved. This provides important practical guidance for risk management in universities.
